# Personalised decision-making in hepatocellular carcinoma: conceptual evolution over two decades

**DOI:** 10.1093/bjsopen/zrag113

**Published:** 2026-08-11

**Authors:** Alessandro Vitale, Gordon Guyatt, Masatoshi Kudo, Laura Kulik, David J Pinato, Laura Crocetti, Edoardo G Giannini, Umberto Cillo

**Affiliations:** Department of Surgical Oncological and Gastroenterological Sciences, Padova University, Padova, Italy; Department of Health Research Methods, Evidence, and Impact, McMaster University, Hamilton, Ontario, Canada; Department of Gastroenterology and Hepatology, Kindai University Faculty of Medicine, Osaka, Japan; Section of Gastroenterology and Hepatology, Department of Medicine, Northwestern Memorial Hospital, Chicago, Illinois, USA; Department of Surgery and Cancer, Imperial College London, Hammersmith Hospital, London, UK; Division of Oncology, Department of Translational Medicine, University of Piemonte Orientale, Novara, Italy; Department of Radiology and Interventional Radiology, Division of Interventional Radiology, University of Pisa, Pisa, Italy; Gastroenterology Unit, Department of Internal Medicine, University of Genova, Genova, Italy; Azienda Ospedaliera Metropolitana IRCCS Policlinico San Martino, Genoa, Italy; Department of Surgical Oncological and Gastroenterological Sciences, Padova University, Padova, Italy

**Keywords:** clinical decision-making, evidence-based medicine, personalized medicine, treatment selection, multidisciplinary care

## Abstract

**Background:**

Evidence-based medicine (EBM) was conceived to support decisions for individual patients by integrating the best available evidence, clinical expertise, and patient values. However, the application of EBM in many fields has become associated with rigid algorithms and guideline pathways, sometimes interpreted as prescriptive rather than as aids to decision-making. Hepatocellular carcinoma (HCC) offers an informative case study of this dichotomy. This paper traces the evolution of personalized decision-making in HCC over two decades and its implications for the future of EBM.

**Methods:**

This is a narrative, conceptual review. Rather than a systematic synthesis, it draws selectively on landmark staging frameworks, guidelines, and methodological literature to construct a schematic account of how clinical reasoning in HCC has evolved.

**Results:**

HCC decision-making has evolved through a sequence of schematic frames: from stage-centred algorithms (the Ptolemaic frame), to recognition of differential treatment effects (the Copernican shift), to multiparametric expert deliberation (the Newtonian perspective), and finally to dynamic, time-dependent, bidirectional strategies (the Einsteinian perspective). The expansion of systemic immunotherapy options has reinforced this bidirectional logic while complicating stage reassessment. This trajectory culminates in a Heisenberg moment, in which uncertainty is acknowledged as intrinsic to individual decisions. The multiparametric therapeutic hierarchy, developed within multidisciplinary HCC practice, reflects this maturation towards a personalized, transparent, context-sensitive approach.

**Conclusion:**

HCC is a paradigmatic example of personalized decision-making in complex, multidisciplinary care. The future of EBM lies not in choosing between algorithms and personalization, but rather in transparent approaches that apply evidence rigorously while acknowledging uncertainty and respecting patient values and local context.

## Teaser

This review uses hepatocellular carcinoma as a case study spanning two decades to trace the conceptual evolution of therapeutic decision-making from rigid stage-based algorithms to multiparametric, dynamic, and uncertainty-aware clinical reasoning. Through a sequence of epistemological frames, the paper shows that personalized decisions require not the elimination of uncertainty, but rather its transparent acknowledgement and management. The multiparametric therapeutic hierarchy is presented as a conceptual framework that reflects this maturation, with implications extending beyond liver cancer to all complex, multidisciplinary surgical disciplines.

## Introduction

Evidence-based medicine (EBM) was conceived as a framework to support clinical decision-making for individual patients by explicitly integrating the best available evidence, clinical expertise, and patients’ values and preferences^1–3^. From its inception, EBM recognized that population-derived evidence cannot be applied mechanically to individuals; rather, clinical judgement is required to interpret it in light of patient-specific circumstances, baseline risk, and uncertainty. EBM was never intended to replace clinical reasoning, but rather to discipline and inform it.

Over time, however, EBM in many clinical fields has sometimes been misconstrued as a strict application of clinical practice guidelines and stage-based algorithms^4–6^.

When EBM is misinterpreted, recommendations originally developed for groups of patients are sometimes treated as rules to be applied to individuals rather than as informed starting points for therapeutic decision-making. The dichotomy between clinical practice guidelines and individual decision-making becomes particularly salient in complex clinical contexts characterized by patient heterogeneity, multiple competing therapeutic options, and substantial uncertainty in the evidence base. Furthermore, when guidelines are applied without regard to patient preferences, they may be misinterpreted by insurers, leading to the denial of an alternative therapy, particularly when the proposed treatment is one normally reserved for an earlier disease stage, a situation sometimes described as ‘treatment stage migration’ towards more radical therapy following reassessment.

Hepatocellular carcinoma (HCC) lends itself as a particularly informative case study in this regard. HCC management has long been organized around stage-based frameworks that combine tumour burden, liver function, and performance status as key clinical parameters to guide treatment selection^4–6^. These multidimensional staging systems represent a significant methodological advance over previous monodimensional systems, explicitly integrating multiple patient- and disease-related dimensions into a coherent classification. Nevertheless, in clinical practice, stage-based recommendations are often interpreted as defining a single preferred treatment pathway, even when several reasonable alternatives exist and when patient-specific factors may substantially modify expected benefits and harms.

With the progressive expansion of therapeutic options for HCC, particularly since the advent of immune checkpoint inhibitor combinations as standard first-line systemic therapy, the limitations of a strictly stage-driven approach have become increasingly apparent^7–9^. A growing body of evidence suggests that no single treatment is appropriate for all patients at a particular clinical disease stage, and that a given treatment may be optimal at different stages^10,11^. This insight has prompted a conceptual shift, in which treatments are no longer viewed as strictly and univocally tied to a single disease stage, but rather coexist as alternatives for patients at a particular disease stage, or even for those at different stages^12–23^. Recognizing the importance of treatment effects does not imply that a specific treatment supersedes the patient as the focus of care; rather, it highlights the need to consider treatment effects alongside patient characteristics, values, feasibility, centre expertise, and context when making individual decisions^1–3^.

At the same time, attempts to move beyond rigid stage-based algorithms have revealed new challenges. Emphasizing treatment effectiveness without adequate attention to patient vulnerability, competing risks, and contextual constraints may lead to inappropriate escalation of care without intended treatment effect^24,25^. This risk underscores a central methodological problem: how to translate population-level evidence and guideline recommendations into transparent, defensible decisions for individual patients when uncertainty is substantial and multiple options are plausible. In addition, rather than committing to a single therapy based on the defined tumour stage, multiple treatment options can lead to more complex decision-making for the patient and their families, highlighting the need for candid, clear-cut discussion by the multidisciplinary team, including palliative care.

The Grading of Recommendations, Assessment, Development and Evaluations (GRADE) approach was developed to address challenges in guideline development by making explicit the judgments that underpin healthcare recommendations^26,27^. Through the evidence-to-decision framework^28,29^, GRADE encourages systematic consideration of benefits and harms, the certainty of evidence, patient values and preferences, feasibility, acceptability, resource use, and equity. These principles provide the methodological context for situating the conceptual evolution described in this paper.

The aim of this review is explicitly conceptual rather than prescriptive. New treatment recommendations are not proposed and a validated decision tool is not presented. Rather, this paper examines successive shifts in decision-making frames in greater depth, using schematic representations and concrete clinical examples to clarify how evidence, uncertainty, and individual patient context are addressed in complex clinical care.

## Ptolemaic frame: stage as the dominant organizing principle

In HCC, therapeutic decision-making has historically been organized around stage-based frameworks, most prominently the Barcelona Clinic Liver Cancer (BCLC) system^4,30,31^. At the time these frameworks were developed, they represented a major advance, integrating multiple patient- and disease-related dimensions (tumour burden, liver function, and performance status) into a single construct. Within this decisional framework, stage serves as the primary organizing principle, and treatment selection is largely determined by stage assignment. Although this approach has improved consistency and transparency in clinical practice, it has also fostered a normative interpretation of staging systems, leading to treatments being perceived as tied to specific stages, with no possibility of modification based on patient factors and the intended goal of therapy tied to each stage.

For each stage, expert panels and guideline developers emphasized a single treatment (or a limited set of treatments) as ‘standard’ or ‘recommended’, typically linking these choices to the results of randomized clinical trials (RCTs). This approach has been adopted and promulgated in major international guidelines^11,32–34^.

In multidisciplinary discussions focused on individual patients, this rigidity may limit consideration of alternative options for those whose clinical profiles do not neatly fit within categorical boundaries, even when they share the same titular stage^31^.

**Figure 1:**
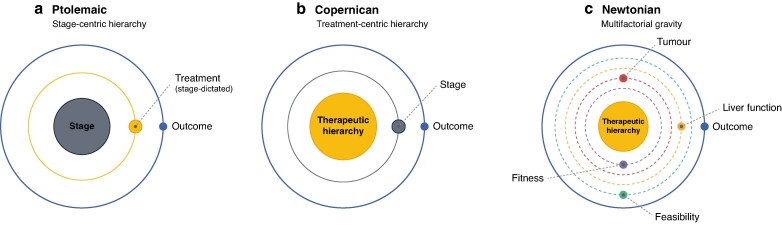
Evolution of HCC treatment decision frameworks: Ptolemaic, Copernican, and Newtonian models The evolution of HCC management reflects the development of scientific paradigms. **a** The Ptolemaic era organizes treatment selection around tumour stage, with interventions following predetermined algorithmic pathways. **b** The Copernican shift recognizes treatment effectiveness as an independent determinant of outcomes, with tumour stage still informing but no longer dictating treatment choice. **c** The Newtonian perspective recognizes that outcomes emerge from the interaction of multiple forces, namely tumour features, liver function, patient fitness, and feasibility, jointly shaping treatment decisions without privileging any single organizing centre. HCC, hepatocellular carcinoma.

*Figure 1a* illustrates this decision-making logic, in which treatment (the Sun) is shown orbiting the stage (the Earth), a simplification that may not serve many patients with HCC well.

## Copernican shift: treatment focused on the patient rather than disease stage

As experience with stage-based algorithms accumulated, discrepancies between predicted prognosis and observed outcomes in HCC became increasingly evident. Patients classified within the same stage often showed markedly different survival trajectories, even when baseline tumour burden and liver function appeared comparable^9,17^. These observations challenged the assumption that tumour stage alone could adequately capture baseline risk, prompting a gradual conceptual shift in the interpretation of prognostic information. These observations also underscore that tumour burden does not fully capture tumour biology.

*Figure 1b* illustrates this conceptual reorientation, in which treatment (the Sun) is placed at the centre of the system. This does not imply that the patient is displaced from the centre of care, but rather that tumour stage (the Earth) alone is insufficient to capture the patient's complexity or dictate treatment choice. Instead, treatment is recognized as having an independent causal effect on outcomes, with a magnitude and direction that depend on multiple patient-specific characteristics.

Rather than abandoning stage-based assessment, this shift reflected a growing recognition that patients at the same stage could benefit from different treatments, and that the same treatment could benefit patients at different stages. Across multiple therapeutic domains, including surgical resection, liver transplantation, and ablative therapies, patients receiving potentially curative treatments consistently showed superior long-term survival compared with those treated with non-curative approaches, even when treatments were applied beyond traditional stage-based indications^12–23,35,36^. This evidence emerged from converging findings across multicentre cohorts, propensity-matched analyses, and comparative observational studies conducted in diverse healthcare systems and populations. RCTs, although ideal, are not attainable or applicable across all facets of HCC therapy, and disregarding data not generated through RCTs is a disservice to patients. Together, these data suggest that, in select patients, more radical or potentially curative treatments may be associated with better survival than stage-assigned non-curative approaches, as summarized in *Table 1*.

**Table 1:** Selected studies illustrating survival evidence across BCLC stages for surgical *versus* non-surgical approaches

Study	Country	Design	No. of patients	BCLC stage	Comparison	Survival outcomes	Analysis
Serper *et al.*^12^	USA	Obs.	3988	All BCLC stages	All treatments *versus* no therapy (reference)	HR: LT 0.18; resection 0.31; ablation 0.50; TACE 0.72; sorafenib 1.70	Multivariable time-varying Cox (BCLC-adjusted)
Vitale *et al.*^36^	Italy	Obs., IPTW	4867	All BCLC stages	All treatments *versus* no therapy (reference)	HR: LT 0.19; resection 0.40; ablation 0.42; TACE 0.58; sorafenib 0.92	Multivariable IPTW Cox (ITA.LI.CA staging-adjusted)
Vitale *et al.*^13^	Italy	Obs.	1196	All BCLC stages	All treatments *versus* LT (reference) at restaging	HR: resection 2.10; ablation 2.93; TACE 3.66; sorafenib 3.57	Multivariable Cox (ITA.LI.CA staging-adjusted) performed at restaging
Kawaguchi *et al.*^14^	Japan, Italy, USA	Obs., IPTW	43 904	All BCLC stages	Resection *versus* ablation *versus* TACE	5-year OS: 46.2% *versus* 33.4% *versus* 27.4%	Multivariable IPTW (tumour-burden adjusted)
Vitale *et al.*^15^	Italy	Obs.	1181	BCLC-stratified	Resection *versus* ablation/TACE	Median OS (months): stage 0, 92 *versus* 62; stage A, 72 *versus* 50; single > 5 cm, 55 *versus* 42; stage B, 52 *versus* 41	Multivariable parametric survival
Kim *et al.*^8^	Korea	Obs.	3515	BCLC-stratified	Surgery or ablation *versus* TACE (stages 0, A, B); surgery or ablation *versus* sorafenib (stage C)	5-year OS: stage 0, 84% *versus* 64%; stage A, 74% *versus* 44%; stage B, 53% *versus* 33%; stage C, 22% *versus* 10%	Multivariable Cox
Vitale *et al.*^35^	Italy	Obs., MAIC	720	BCLC-A oligonodular	Resection *versus* ablation *versus* TACE	5-year OS: 56.4% *versus* 39.9% *versus* 29.2%; HR 1.41 (95% c.i. 1.07, 1.81) and 1.86 (95% c.i. 1.29, 2.68)	MAIC and weighted multivariable Cox
Pecorelli *et al.*^9^	Italy	Obs., PSM	485	Intermediate (BCLC-B)	Curative *versus* TACE *versus* sorafenib *versus* BSC	Median OS (months): 45 *versus* 30 *versus* 14 *versus* 10; HR curative *versus* BSC: 0.20 (95% c.i. 0.10, 0.40)	PSM + multivariable Cox
Yin *et al.*^17^	China	RCT	173	Intermediate (BCLC-B)	Resection *versus* TACE	3-year survival: 51.5% *versus* 18.1%; HR 0.43 (95% c.i. 0.29, 0.64); *P* < 0.001	Randomized trial (open-label, single-centre)
Mazzaferro *et al.*^18^	Italy	RCT	74	Intermediate (after downstaging)	LT *versus* non-transplant	5-year OS: 77.5% *versus* 31.2%; HR 0.32 (95% c.i. 0.11, 0.92), *P* = 0.035	Phase 2b/3 RCT
Kokudo *et al.*^19^	Japan	Obs., PSM	2116	Advanced (portal vein invasion)	Resection *versus* non-surgical	Median OS (years): 2.45 *versus* 1.57; HR 1.43 (95% c.i. 1.25, 1.63), *P* < 0.001	PSM + multivariable Cox
Kokudo *et al.*^20^	Japan	Obs., PSM	446	Advanced (hepatic vein invasion)	Resection *versus* non-surgical	Median OS (years): 3.42 *versus* 1.81; HR 1.61 (95% c.i. 1.30, 2.01), *P* < 0.001	PSM + multivariable Cox
Mei *et al.*^21^	China	Obs., PSM	144	Advanced (macrovascular invasion)	Resection *versus* sorafenib	Median OS (months): 27.2 *versus* 13.0; *P* < 0.001	PSM survival analysis
Govalan *et al.*^22^	USA	Obs., PSM	264	Advanced (macrovascular invasion)	Resection *versus* systemic therapy	Median OS (months): 21.4 *versus* 8.1; *P* < 0.001	PSM survival analysis
Famularo *et al.*^23^	Italy	Obs., IPTW	478	Advanced (portal vein invasion)	Resection *versus* sorafenib	5-year OS: 55.9% *versus* 12.8%; HR 4.44 (95% c.i. 3.19, 6.15), *P* < 0.001	IPTW multivariable Cox

This is not a systematic review; only illustrative studies are presented. BCLC, Barcelona Clinic Liver Cancer; Obs., observational; HR, hazard ratio; LT, liver transplantation; TACE, transarterial chemoembolization; IPTW, inverse probability of treatment weighting; ITA.LI.CA, Italian Liver Cancer; OS, overall survival; MAIC, matching-adjusted indirect comparison; c.i., confidence interval; PSM, propensity score matching; BSC, best supportive care; RCT, randomized clinical trial.

The recognition that patients at the same clinical stage may benefit from different treatments grounds decisions on patient-specific characteristics, baseline risk, co-morbidities, and preferences^1–3^. The Copernican shift reflects the need to make causal reasoning explicit. Although patient and stage characteristics define the clinical context in which decisions are made, the effect of treatment on outcomes cannot be inferred solely from these characteristics. It must consider patient characteristics as defining subgroups in which treatment effects will differ.

The long-standing use of transarterial chemoembolization (TACE) in intermediate-stage HCC illustrates the need for individualized treatment. For nearly two decades, clinicians have regarded TACE as the standard of care for BCLC-B disease, largely based on early small randomized trials showing a survival benefit over best supportive care^37,38^. However, TACE is not curative. Observational studies have consistently reported findings suggesting that patients with limited tumour burden (those most likely to benefit from TACE) may also be suitable candidates for potentially curative treatments^39^. In contrast, those with extensive disease may derive limited benefit from TACE^40^.

Comparative observational evidence suggests that when intermediate-stage patients are first assessed for suitability for potentially curative therapies, those who can safely undergo such treatments achieve survival outcomes not attainable with TACE alone^9,17^. This finding does not hinge on defining a narrow subgroup; rather, it reflects a shift in decision logic: exploring candidacy for curative options before defaulting to stage-assigned therapies.

This experience highlights a broader conceptual limitation: when randomized evidence addresses only a subset of relevant comparisons, exclusive reliance on trial design rather than on the totality of evidence may constrain, rather than support, individualized decision-making. Consistent with GRADE principles, high-quality observational evidence can and should complement randomized trials when addressing questions of treatment suitability and expected benefit in heterogeneous populations^41^.

These observations revealed a limitation of rigid algorithmic reasoning. Stage-based recommendations implicitly assume that treatment effectiveness is homogeneous across stages and often interpret deviations from recommended pathways as inappropriate care.

Acceptance of this paradigm by insurers disadvantages patients who can derive significant benefit outside a stage-based therapeutic system. When applied more broadly to clinical practice, treatment effects may vary substantially among patients classified within the same stage, reflecting not only differences in tumour and patient characteristics but also contextual factors such as technical feasibility, centre experience, and operator expertise^25^.

Accumulating evidence in HCC has challenged this assumption, showing that treatment effects vary substantially among patients at the same stage^12–23,35,36^. This recognition led to the idea that some treatments are more effective than others. However, it still recognizes that this gradient of effectiveness is not universal across patients: it will be true for some patients but not others^24^. However, the Copernican reframing also revealed an important limitation. While recognizing that tumour stage alone is insufficient to guide treatment choice, this perspective does not explicitly identify which patient- and context-related domains condition treatment effectiveness. By placing treatment at the centre without making these modifying factors explicit, the framework risks oversimplification, particularly when applied without sufficient clinical expertise. In such circumstances, emphasizing treatment effectiveness in isolation may inadvertently encourage inappropriate escalation of care and overtreatment. This limitation highlights the need for a more explicit, multiparametric (that is, the simultaneous consideration of multiple patient and contextual characteristics, and how these modify the expected benefits, harms, and suitability of different treatment options) representation of clinical complexity and contextual constraints, thereby motivating a transition towards a Newtonian perspective^25^.

## Newtonian perspective: explicit judgment under irreducible complexity

Recognizing the limitations of both stage-centred and treatment-centred frames leads to a third perspective in which clinical decision-making is understood as the outcome of multiple interacting determinants rather than a single dominant organizing principle. In this Newtonian view, outcomes are shaped by the interplay of baseline risk, tumour biology, liver function, co-morbidities, patient values and preferences, feasibility, resource constraints, and the anticipated benefits and harms of treatment^25,42^.

At first glance, this perspective may seem extraordinarily complex, unsustainable, and beyond individual cognitive capacity. However, the Newtonian frame does not assume that all relevant variables must be considered simultaneously or exhaustively. Rather, it reflects how clinicians routinely manage complexity by prioritizing the most consequential factors for a given patient, recognizing that different domains become salient in various clinical contexts^1,2^. For example, in some patients, liver function may be the dominant constraint limiting otherwise effective treatments (for example, patients with decompensated liver cirrhosis); in others, tumour extent or biology may outweigh competing considerations (for example, patients with extrahepatic metastases or very elevated α-fetoprotein levels); in still others, patient values and preferences may appropriately take precedence when potential benefits are modest and treatment burden substantial (for example, patients with particular religious beliefs, such as Jehovah’s Witnesses). Complexity is therefore addressed through structured reduction and transparent prioritization, not comprehensive optimization.

Multidisciplinary discussion plays a central role in this process by bringing to bear the expertise and insights of individuals addressing the decision-making process from different perspectives. Although empirical evidence on how multidisciplinary conferences function and influence outcomes remains limited and heterogeneous, multidisciplinary teams (MDTs) are best understood conceptually as forums in which diverse perspectives interpret available evidence and make explicit the judgments, trade-offs, and uncertainties underlying individual decisions^28,43^. Importantly, multidisciplinary deliberation does not eliminate subjectivity or variation; instead, it renders transparent how evidence is weighed and contextualized, thereby making these judgments open to scrutiny and accountability.

This Newtonian perspective does not rely on intuition, wisdom, or other forms of reasoning beyond logic or rationality. On the contrary: it emphasizes explicit judgement under uncertainty and fully involves the patient in the decision-making process. Decisions emerge from the reasoned consideration of imperfect evidence, contextual constraints, and the patient’s values and preferences, as elicited and interpreted in clinical encounters. Within this framework, different clinician–patient dyads may reasonably reach different conclusions when faced with the same information, not because evidence-based practice has failed, but because individual patients may value benefits, harms, and risks differently^1,28^. This way of organizing clinical reasoning is illustrated schematically in *Fig. 1c*, in which multiple interacting forces jointly shape decision-making without privileging any single organizing centre.

The Newtonian frame aligns closely with the foundational principles of EBM^28,43^. Both recognize that evidence alone is insufficient to determine optimal decisions for individuals and that explicit judgments are required to translate evidence into action. Rather than seeking to eliminate complexity or replace judgment with algorithms, this perspective aims to render the reasoning process transparent, structurally reproducible, and open to critical appraisal.

Recent international guidelines, including those from the European Association for the Study of the Liver (EASL)^44^, the European Society for Medical Oncology (ESMO)^45^, and the American Association for the Study of Liver Diseases (AASLD)^46^, strongly endorse the role of MDTs in HCC decision-making and recommend MDT discussion for all patients with HCC. However, these recommendations define a process of care rather than a specific intervention, reflecting the need for consensus-based judgment intrinsic to guideline development, regardless of the certainty of the evidence^47^.

MDTs should not be understood as decision-making algorithms or interventions that resolve clinical complexity. Rather, they provide an institutional setting for the explicit consideration of multiple patient-specific factors that inform decisions. MDT discussions make clinicians’ assumptions, trade-offs, and sources of uncertainty visible, but they do not eliminate subjectivity or guarantee superior decisions.

This discussion neither proposes nor validates a specific MDT framework. Instead, MDTs are treated as the context in which Newtonian reasoning that recognizes patient complexity typically occurs. The effectiveness of various MDT structures, processes, and decision-support tools remains an empirical question requiring formal evaluation.

## Einsteinian perspective: time, dynamics, and sequential treatment decisions

The Newtonian evolution of decision-making in HCC recognized that optimizing treatment requires consideration of multiple patient characteristics and therapeutic options. However, this multiparametric perspective often treats decisions as occurring at a single point in time, implicitly assuming that a baseline assessment adequately captures the relevant clinical context. In clinical practice, both in HCC and in many other areas of oncology and chronic disease, treatment decision-making is inherently dynamic and unfolds over time, as patient characteristics, tumour biology, organ function, and treatment effects evolve^48^. As such, the initial goal of therapy may pivot over time. This recognition marks a further conceptual shift, in which time becomes an explicit dimension of decision-making. In this perspective, treatment choice is no longer a one-off event anchored to an initial stage assignment but part of a longitudinal process in which earlier interventions modify subsequent prognosis and therapeutic opportunities. This temporal framing does not imply increased complexity for its own sake; rather, it reflects the reality that treatments can alter disease trajectories in ways not captured by static baseline classifications.

Advances in HCC management have progressively reinforced this temporal dimension. Improvements in locoregional and systemic therapies and in the care of patients with chronic liver disease have, in select patients, enabled reductions in tumour burden, stabilization or improvement of liver function, and changes in performance status over time, thereby altering both prognosis and eligibility for subsequent treatments^49–56^. Concepts such as stage migration, downstaging, and conversion therapy emerged from these observations, not as departures from guideline-based care but as descriptive labels for clinically observed changes in trajectory following previous treatment.

Crucially, recognizing downstaging does not imply that more intensive therapy is universally appropriate or that escalation is always beneficial. Instead, it reflects the observation that, in carefully selected patients, intermediate or systemic treatments may enable access to subsequent options that were previously infeasible. In this sense, downstaging is not a goal in itself but a possible treatment consequence that, if realistic, must be considered by weighing risks, competing prognostic factors, and patient preferences^44^.

The advent of immune checkpoint inhibitor combinations as first-line systemic therapy^57,58^ has accentuated, rather than created, this temporal logic. Regimens such as atezolizumab plus bevacizumab and durvalumab plus tremelimumab have improved response rates and long-term survival compared with tyrosine kinase inhibitor monotherapy, and a subset of patients now achieves deep and durable radiological responses^44^. These responses have made downstaging and conversion to potentially curative resection or transplantation realistic considerations for patients previously regarded as candidates for palliative systemic treatment alone, thereby reinforcing the bidirectional pathways described above^48^. At the same time, immunotherapy has complicated the logic of stage reassessment: response is often heterogeneous and asynchronous across tumour sites, the relationship between radiological response and survival benefit is imperfect, and decisions about whether (and when) to pivot from systemic therapy towards a locoregional or surgical option must increasingly be made under conditions of evolving, incompletely characterized evidence^39^. Thus, the expanding systemic landscape does not restore a stage-anchored decision sequence; it amplifies the need to treat stage, eligibility, and therapeutic intent as dynamic, time-dependent states rather than fixed properties.

Within this dynamic framework, the traditional unidirectional flow of stage-based algorithms, from early to advanced disease and from curative to palliative intent, has proved inadequate for capturing real-world decision-making. For some patients, treatment pathways can be bidirectional: patients initially unsuitable for curative therapies may, after effective intermediate or systemic treatment, become candidates for resection or transplantation, whereas others may appropriately transition in the opposite direction as disease or liver function deteriorates.

**Figure 2:**
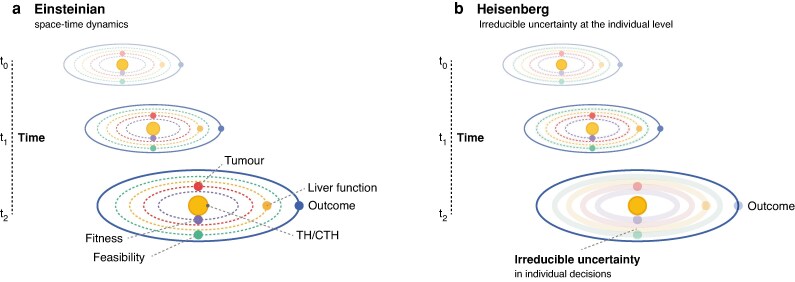
Evolution of hepatocellular carcinoma treatment decision frameworks: Einsteinian and Heisenberg perspectives **a** In the Einsteinian model, the introduction of time as an explicit dimension of decision-making allows bidirectional movement through the therapeutic hierarchy, recognizing that earlier interventions modify subsequent prognosis and therapeutic opportunities. **b** Although the underlying dimensions of clinical reasoning remain the same, the Heisenberg perspective captures the irreducible uncertainty that characterizes individual decisions. The blurred orbits convey the conceptual point that patient trajectories and contextual factors cannot be fully resolved at the individual level; only the observed outcome is ultimately determined. The goal of methodological frameworks is therefore not to eliminate uncertainty but to make it explicit and transparent. TH, therapeutic hierarchy; CTH, converse therapeutic hierarchy.

*Figure 2a* illustrates the time-dependent and bidirectional organization of treatment decisions. Rather than depicting a hierarchy of preferred treatments, the figure presents treatment as a sequential process in which interventions modify patient states over time, thereby expanding or constraining future options.

This perspective does not negate the relevance of tumour stage, baseline prognosis, or assessment that considers the full range of relevant patient characteristics. Instead, it relativizes their significance by placing them within a temporal continuum. Stage, risk profile, and treatment eligibility are treated not as immutable properties but as context-dependent states that may change in response to previous therapeutic interventions.

This shift challenges the implicit assumption that guideline recommendations are applied at a single decision point. It underscores the need for decision frameworks that can accommodate sequential, adaptive choices, in which treatment effectiveness is evaluated not only by immediate outcomes but also by its capacity to modify future therapeutic opportunities and long-term trajectories^49,59,60^.

The Einsteinian evolution thus completes the transition from static, stage-based reasoning to a dynamic, process-oriented view of HCC management. At the same time, by explicitly incorporating time and sequence, it renders uncertainty about future states and outcomes unavoidable, thereby setting the stage for the subsequent Heisenberg perspective on personalized decision-making.

## Heisenberg moment: irreducible uncertainty and individual clinical decisions

The incorporation of time and bidirectionality into HCC management reveals a further conceptual boundary: uncertainty is not a residual imperfection in the evidence base but an intrinsic feature of clinical decision-making^31,39^. Once treatment choices are understood as sequential and adaptive, uncertainty becomes inseparable from the act of choosing. Treatment selection does not merely apply evidence to a static clinical state, it actively shapes subsequent prognosis, therapeutic opportunities, and future decision spaces. As a result, uncertainty cannot be eliminated through finer stratification or increasingly complex algorithms; it must instead be explicitly acknowledged and managed. *Figure 2b* illustrates this epistemological limit.

These challenges are particularly evident in HCC, where many clinically relevant decisions rely on observational evidence, indirect comparisons, and possible subgroup effects. Even when tumour characteristics, liver function, and patient fitness are carefully assessed, clinicians often need to make decisions in which the anticipated benefits and harms are informed by evidence with low or very low certainty^1–3^.

## From population-level recommendations to individual decisions: role of GRADE

The GRADE Working Group developed a rigorous framework for translating evidence into healthcare recommendations^28,43^. Although GRADE is most widely applied in guideline development and health technology assessment, it is grounded in the foundational principle that recommendations are intended to inform, rather than replace, decisions made for individual patients. The majority of GRADE recommendations are conditional, explicitly acknowledging uncertainty, variability in patient values, and the need for individualized deliberation.

Conditional recommendations are particularly relevant in HCC, where uncertainty is the norm rather than the exception. Rather than aiming to eliminate uncertainty, GRADE offers a structured way to make it visible: what is known with reasonable confidence, what remains uncertain, and where value judgements enter the reasoning process^28,43^. This perspective is fully compatible with the recognition that, in complex clinical conditions, the challenge is not the absence of rigorous methodology but the consistent application of its principles to individual cases.

## Prognosis, PROGRESS, and the conceptual position of multiparametric therapeutic hierarchy

**Figure 3:**
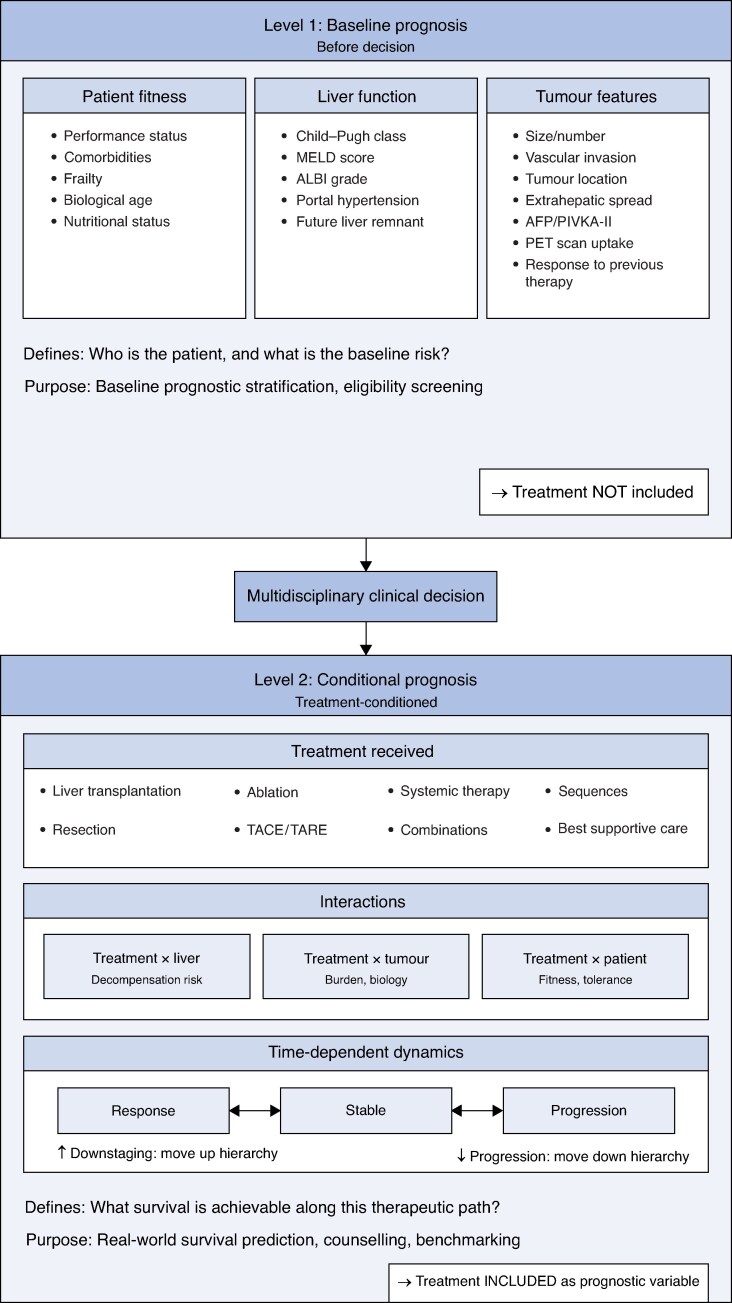
Two-level prognostic framework integrating treatment decisions in hepatocellular carcinoma Prognostic assessment in hepatocellular carcinoma can be viewed as a two-tier process. Level 1 (baseline prognosis) is established before treatment allocation and integrates patient fitness, liver function, and tumour features, including biological markers and response to previous treatments. This level supports initial risk stratification and eligibility assessment, but does not include treatment. Level 2 (conditional prognosis) is determined after clinical decision-making and incorporates treatment as a variable influencing prognosis (together with its interactions with patient, liver, and tumour characteristics) with dynamic factors over time, such as response, downstaging, conversion, and progression. This framework aligns with the PROGRESS guidance by differentiating baseline from treatment-conditioned prognosis and situates the multiparametric therapeutic hierarchy at the interface between these two levels. MELD, Model for End-stage Liver Disease; ALBI, albumin–bilirubin score; AFP, α-fetoprotein; PIVKA-II, protein induced by vitamin-K absence II; PET, positron emission tomography; TACE, transarterial chemoembolization; TARE, transarterial radioembolization.

Prognostic assessment in HCC can be conceptualized as a two-level process (*Fig. 3*). The first level concerns the baseline prognosis, established before treatment allocation and informed by patient fitness, liver function, tumour features, and biological markers. This level supports initial risk stratification and eligibility assessment but does not include treatment effects. The second level concerns conditional prognosis, determined after clinical decision-making and explicitly incorporating treatment as a variable influencing outcomes, along with its interactions with patient characteristics and dynamic factors over time, such as response, downstaging, conversion, and progression.

**Figure 4:**
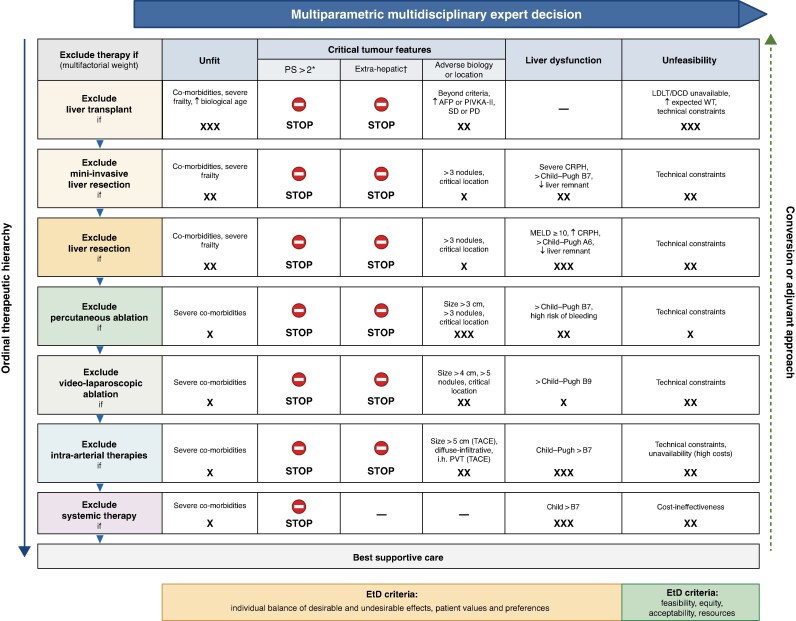
MTH framework for hepatocellular carcinoma The left vertical axis represents the ordinal therapeutic hierarchy, ranking treatments from liver transplantation to best supportive care by expected survival benefit under favourable conditions. The horizontal axis represents the multiparametric assessment of contraindications across four domains: patient fitness, critical tumour features, liver dysfunction, and unfeasibility. For each treatment option, the weight of each variable as a relative contraindication is indicated as follows: —, irrelevant; X, low; XX, intermediate; XXX, relevant; STOP, absolute contraindication. The dashed right vertical axis represents the converse therapeutic hierarchy, in which effective conversion or neoadjuvant strategies may enable access to hierarchically superior treatments. The two coloured bars beneath the grid indicate the conceptual alignment between MTH domains and established criteria for individualized clinical decision-making: patient fitness, critical tumour features, and liver dysfunction jointly capture the individual balance of desirable and undesirable effects, together with patient values and preferences, whereas unfeasibility encompasses feasibility, equity, acceptability, and resource considerations. *PS reflects tumour-related symptoms. †Extrahepatic metastases, main portal vein, or inferior vena cava invasion. PS, performance status; AFP, α-fetoprotein; PIVKA-II, protein induced by vitamin-K absence II; SD, stable disease; PD, progressive disease; LDLT, living donor liver transplantation; DCD, donor after circulatory death; WT, waiting time; CRPH, clinically relevant portal hypertension; MELD, Model for End-stage Liver Disease; TACE, transarterial chemoembolization; i.h., intrahepatic; PVT, portal vein thrombosis; EtD, evidence to decision. Adapted from Trevisani *et al*.^31^.

This distinction aligns with the PROGRESS framework, which differentiates fundamental prognosis from prognostic factors, prognostic models, and stratified medicine^61–64^. Within this landscape, the multiparametric therapeutic hierarchy (MTH) can be situated at the interface between baseline and treatment-conditioned prognosis^25,31^. Originally developed as a clinical response to the rigidity of stage-based algorithms, MTH treats treatment eligibility as a conditional prognostic factor and integrates multiple prognostic dimensions into a multiparametric structure (*Fig. 4*).

In this sense, MTH is not proposed here as a new staging system, a decision algorithm, or a validated decision-support tool. It is presented as a conceptual framework that reflects how multidisciplinary clinical reasoning in HCC has evolved to integrate baseline prognosis, treatment selection, and dynamic reassessment within a single structure. The extent to which MTH aligns with established methodological frameworks for individualized decision-making, and how such alignment might be operationalized, warrants separate methodological treatment.

## Convergence with the BCLC 2026 complexity, uncertainty, subjectivity, and emotion framework

The 2026 update to the BCLC system introduces the complexity, uncertainty, subjectivity, and emotion (CUSE) framework as an explicit acknowledgement that treatment decision-making in HCC cannot be fully captured by rigid stage-based algorithms^39^. This marks an important and timely convergence with broader efforts to recognize uncertainty and individualization in clinical care.

CUSE and methodologically grounded approaches to individualized decision-making share substantial common ground. Both recognize that uncertainty is unavoidable, that individual values matter, and that algorithms cannot replace clinical judgement. Differences between the two frameworks lie less in their underlying goals than in their intellectual lineage. CUSE draws primarily on descriptive models of illness uncertainty and clinical experience, whereas formal methodological frameworks developed within the EBM tradition provide structured procedures for translating evidence into transparent recommendations open to critical appraisal. Rather than viewing these approaches as competing, they can be understood as complementary. CUSE articulates why personalization is necessary; methodological frameworks rooted in EBM offer ways to structure personalization so that it remains transparent, proportionate, and accountable. Importantly, neither CUSE nor MTH has been prospectively validated as a decision-support intervention, and both should be regarded as conceptual contributions rather than prescriptive solutions.

As illustrated in *Fig. 4*, MTH organizes treatment options along an ordinal hierarchy defined by expected survival benefit while simultaneously accounting for a multidimensional assessment of patient fitness, tumour features, liver function, and contextual constraints. This structure reflects the clinical intuition that decision-making in HCC cannot be reduced to a single axis, and its alignment with established criteria for individualized decision-making, illustrated conceptually at the base of *Fig. 4*, suggests that clinical reasoning in complex multidisciplinary contexts has converged on principles already articulated within the methodology of EBM.

The Heisenberg moment thus underscores a fundamental insight: uncertainty cannot be eliminated from clinical decision-making, whether decisions are strongly personalized or more guideline-concordant. The goal of methodological frameworks is therefore not to achieve perfect certainty but to make explicit what is known, what is uncertain, and where value judgments enter the decision-making process. In highly individualized contexts such as HCC, this transparency, rather than algorithmic optimization, represents the optimal extension of EBM.

## Beyond algorithms: towards authentic EBM

The Heisenberg perspective highlights the conceptual limits of algorithmic certainty in individual clinical decision-making. As decisions become increasingly individual, sequential, and sensitive to patient values and context, further refinement of static algorithms yields diminishing returns. Therefore, the central methodological challenge is to recognize that certainty itself lies on a continuum and to make explicit where a given decision falls on it.

What appears to be lacking is not a conceptual framework for individualized decision-making, which is already implicit in EBM, but the consistent and transparent translation of this reasoning into everyday clinical practice. Integrating anticipated benefits and harms, patient values and preferences, contextual constraints, and temporal dynamics imposes substantial cognitive demands on clinical teams, particularly in complex and heterogeneous conditions such as HCC. Digital tools and decision-support systems may, in the future, contribute to making such reasoning more transparent and reproducible, provided they are designed to support rather than replace clinical judgment^65–68^.

HCC provides a particularly instructive testing ground for this conceptual evolution. Its marked clinical heterogeneity, reliance on multidisciplinary expertise, frequent use of sequential and bidirectional treatment strategies, and persistent uncertainty despite an expanding evidence base make it emblematic of challenges increasingly encountered across modern multidisciplinary medicine. However, the implications extend well beyond HCC. The issues raised here, namely how to translate evidence into decisions under uncertainty, how to integrate values and context transparently, and how to avoid both rigid algorithms and unstructured intuition, are common across many areas of contemporary clinical care.

The future of EBM does not lie in choosing between algorithms and personalization. This account, it should be acknowledged, has important limitations: it is a narrative, conceptual review rather than a systematic one, drawing selectively on landmark staging frameworks, guidelines, and methodological literature chosen to illustrate a conceptual trajectory, with no claim to comprehensive or reproducible coverage of the evidence, and it is not intended to function as a clinical algorithm or to prescribe specific treatment choices. With these caveats in mind, the findings suggest that the way forward lies in developing transparent, proportionate, and methodologically honest approaches that enable clinicians to apply evidence rigorously while acknowledging uncertainty and respecting individual patient context. HCC is not an exception in this regard but a paradigmatic example of the tensions and opportunities that define personalized decision-making in the era of complex, multidisciplinary care.

## Data Availability

This is a conceptual analysis paper. No original data sets were generated or analysed during this study. All data supporting the arguments presented are derived from published sources cited in the reference list.
